# Perinatal Suicide

**DOI:** 10.1111/jmwh.13738

**Published:** 2025-01-18

**Authors:** Pamela J. Reis

**Affiliations:** ^1^ East Carolina University College of Nursing Greenville North Carolina

## PERINATAL SUICIDE

The tragedy of preventable perinatal deaths among birthing people continues to take its toll on our nation. This includes death by suicide during the perinatal period as a profound and leading cause of maternal mortality. Mental health disorders are the leading cause of maternal mortality in the United States according to the most recent data from the Centers for Disease Control and Prevention (CDC).^1^ The CDC defines deaths due to mental health conditions as those because of suicide, overdose, or drug poisoning related to substance use disorder (SUD), and other deaths determined by morbidity and mortality review committees to be related to a mental health condition, including SUD.^2^ Suicide during the perinatal period accounts for approximately 7% of deaths during pregnancy and 20% of postpartum deaths, shockingly surpassing death by postpartum hemorrhage or hypertensive disorders.^3^ The purpose of this commentary is to highlight current literature in perinatal suicide and to provide guidance and resources for clinicians.

## DEFINITION OF TERMS ASSOCIATED WITH PERINATAL MORTALITY

Based on a review of the literature, Chin et al^3^ provided the following definition of terms associated with perinatal mortality:

**Pregnancy‐associated death**: Death during pregnancy or within one year of the end of pregnancy, irrespective of cause.
**Pregnancy‐related death**: Death during pregnancy or within 1 year of pregnancy from a pregnancy complication, a chain of events initiated by pregnancy, or the aggravation of an unrelated condition by physiologic effects of pregnancy.
**Maternal death**: Death during pregnancy or within 42 days of the end of pregnancy, from any cause related to or aggravated by pregnancy or its management, but not from accidental or indirect causes.
**Late maternal death**: The death of a [pregnancy‐capable person] from direct or indirect obstetric causes more than 42 days but less than 1 year after termination of pregnancy.^3^ ^(Box 1)^

Pregnancy‐related deaths because of mental health conditions are described as any death due to a maternal health condition, such as depression or other psychiatric illnesses and SUD and drug overdose (intentional or not intentional). Death by suicide includes unintentional and accidental drug overdose, as well as instances for which the intent to die by suicide is known.^2^

It is not uncommon for mental health disorders such as depression, anxiety, and bipolar disorder to begin or worsen during pregnancy and the postpartum period.^4^ The spectrum of suicide disorders is more prevalent among birthing people with a history of depression or bipolar disorder.^4^ The *Diagnostic and Statistical Manual of Mental Disorders, Fifth Edition* (*DSM‐5*) published in 2013, introduced suicidal behavior disorder (SBD) under conditions for further study, defining SBD as a self‐initiated sequence of behaviors leading to one's own death within the previous 24‐month period.^5^ Unfortunately, the clinical usage of the definition of SBD for predicting death by suicide has not resulted in a decrease in suicide, and the diagnosis and manifestations of SBD and its association with suicidal ideation and other self‐harming behaviors is unclear. The American Psychiatric Association's latest release, the *DSM‐5‐Text Revision*, published in 2022, did not elaborate on the SBD diagnosis in a manner that clinicians and researchers found especially useful, and was ultimately moved from Conditions for Further Study to Other Conditions That May Be a Focus of Clinical Attention. The rationale for this change was that suicide did not strictly meet the criteria for a mental health disorder, but instead was a behavior with diverse causes.^5^

## UNDERSTANDING THE SCOPE OF THE PROBLEM

Determining the incidence of perinatal death because of suicide is challenging, and research is evolving to understand risks and possible prevention of this catastrophic outcome. The CDC extracts data from International Statistical Classification of Diseases codes to determine underlying causes of perinatal death. Only recently has perinatal suicide, as well as deaths from drug overdose or poisoning, been included in pregnancy‐related maternal death counts. Although increased vigilance to identify perinatal suicide has improved, outcomes indicate that the reporting of maternal death by suicide increased significantly when the definition of perinatal was extended to 1 year postpartum.^6^

Death certificates present an ongoing challenge in identifying suicide as a cause of death for people in the perinatal period. Reporting errors are routinely identified despite the 2003 revision of the US Standard Certificate of Death that added a pregnancy check box to death certification.^7^ Identification of death by suicide often requires additional surveillance such as autopsy, postmortem pregnancy tests, and outpatient mental health records.^3^

Racial and ethnic differences in mortality because of perinatal suicide can be difficult to quantify because of small samples and the tendency to classify some races or ethnicities (such as Native American) as other.^3^ Underreporting significantly impacts the collection of these important demographic data. Research suggests, however, that Black, non‐Hispanic women have a higher risk for suicide than other races and ethnicities.^3^ It has been observed that women who report their race as other are approximately 3 times more likely than White individuals to report suicidal ideation in the postpartum period.^8^

## POSTPARTUM PERIOD AS A RISK FACTOR FOR SUICIDE

It has been previously suggested that pregnancy, birth, and the postpartum period induces feelings that are protective against suicidal ideation. However, the developing research in perinatal suicide negates this belief. Chin et al^3^ examined the prevalence and correlates of suicidal behaviors through a review of current literature that specifically focused on maternal suicide. Overall, the authors found that the prevalence of death by suicide during the perinatal period varied, with reports of greater incidences of suicidal behavior during the second and third trimesters. According to the literature reviewed, Chin et al^3^ found that most suicides occur late in the perinatal period, between 43 and 365 days after the pregnancy ended. Severe mental health disorders after birth and a history of self‐harm were noted to be high risk factors for suicide in the postpartum period.^3^ The postpartum period is a particularly high‐risk period for suicide. It is estimated that up to 75% of all perinatal suicide deaths occur between 6 weeks to 1 year after giving birth.^3^ Chin et al^3^ observed in their review of the literature that non‐Hispanic Black women were at highest risk for suicidal thoughts and intent.

## SCREENING FOR PERINATAL SUICIDE

Screening for depression, anxiety, and other perinatal mood disorders has been established as best practice and evidence‐based clinical care. Yet there remains lack of consensus about routine screening for suicide. In June 2023, the US Preventive Services Task Force issued a recommendation on depression and suicide screening for all adults. Although screening for depression was recommended for all adults, the task force concluded that the current evidence is insufficient to assess the balance of benefits and harms of screening for suicide risk in the adult population, including pregnant and postpartum persons. ^9^ Both the Patient Health Questionnaire (PHQ) and the Edinburgh Pregnancy/Postnatal Depression Scale include one question about suicide thoughts. Having thoughts of suicide, however, does not necessarily mean that an individual is at imminent risk of death by suicide, making the screening process more challenging. Suicidal ideation consists of intrusive thoughts and contemplations and preoccupation with death and suicide. Passive suicidal ideation are thoughts of worthlessness and death, but without a plan to end one's own life. Active suicidal ideation is thoughts of suicide with a plan or intent to harm oneself.^10^

Well‐known risks for completed suicide during the perinatal period are: a personal or family history or current diagnosis of depression or anxiety disorder, psychiatric hospitalization, an abrupt discontinuation of psychotropic drugs, history of suicidal ideation and suicide attempts, SUD (prior or current), pregnancy loss, unplanned pregnancy, limited education attainment, low income household, intimate partner abuse, history of adverse childhood experiences including rape, low social support, and age 40 years and older or age less than 20 years.^4^

## PERINATAL SUICIDE POLICIES AND PREVENTION STRATEGIES

Zero Suicide is a 7 element, transformational safe care model first developed by the 2012 National Strategy for Suicide Prevention. The premise of this model is that all individuals who encounter a health care provider should be screened for suicide risk. The Zero Suicide model and framework was developed through the Educational Development Center, a nonprofit organization that promotes lasting solutions to improve education, health, and economic opportunity.^11^ Zero Suicide was adopted as a priority for the National Action Alliance for Suicide Prevention and the Suicide Prevention Resource Center, a project of the Educational Development Center. The purpose of Zero Suicide is to empower behavioral health care systems and all entities that provide care to individuals with behavioral health needs with the most effective, data‐informed, and evidence‐based suicide care practices available. The model recommends that systems adopt a zero‐based mindset by routinely and consistently using evidence‐based practices focused on patient safety and hope and recovery for people at risk for suicide. The model reinforces that asking directly about suicide and responding appropriately should be as routine as having vital signs obtained at every health care visit.^11^ The 7 elements of the model are (1) leading system‐wide change committed to reducing suicide, (2) training a competent and compassionate workforce, (3) identifying at‐risk individuals through comprehensive screening and assessment, (4) engaging all individuals at risk for suicide using a suicide care management plan, (5) treating individuals at risk for suicide using evidence‐based treatments and strategies, (6) transitioning individuals through connecting them with supportive contacts, and (7) improving policies and processes through continuous quality improvement. Individual organizations use the Zero Suicide Framework and toolkit to develop customized suicide identification and awareness programs based on their populations and communities.

Research evidence about the efficacy of Zero Suicide Framework is scant and still evolving. Stapelberg et al^12^ evaluated the Zero Suicide Framework after implementation in a large mental health service in Australia. The authors examined the incidence of repeated suicide attempts and found a reduction in the number of repeated suicide attempts and a longer period to a subsequent attempt for individuals receiving care using the Zero Suicide Framework. Summaries of current research evaluating the efficacy of the Zero Suicide model are listed on the Zero Suicide website.^11^

September is recognized annually as National Suicide Prevention Month. On September 10, 2024, the Benjamin Miller Policy Center for Maternal Mental Health and the American Foundation for Suicide Prevention hosted a Congressional Briefing on Maternal Suicide on World Suicide Prevention Day to highlight suicide as the leading cause of maternal mortality. Policy recommendations discussed during the briefing were as follows.

### Recommendations to Congress^13^

1. Expansion of funding for state Maternal Mortality Review Committees and Perinatal Quality Committees to implement the Zero Suicide Framework.
2. Creation of a Federal Maternal Psychiatry Consultation Program to permanently fund existing state programs and to expand programs to other states, to be funded through the Health Resources and Services Administration.
3. Revision of the Child Welfare Law to remove mandatory reporting to Child Protective Services in favor of safety plans when a patient is suicidal.

### Recommendations to the Centers for Medicare and Medicaid Services^13^

1. Require states to report the Healthcare Effectiveness Data and Information Set Perinatal Screening Measures.
2. Require measures for suicide screening and assessment and safety plan development in primary health care and perinatal care settings.
3. Evidence‐based interventions such as cognitive behavioral therapy, dialectical behavioral therapy, and collaborative assessment and management of suicidality, which is an intensive, suicide‐specific therapy, are recommended to target suicide risk among individuals.

## SCREENING FOR SUICIDE RISK: CALL TO ACTION

In addition to using well‐known depression and anxiety screening tools such as the Edinburgh Perinatal Depression Scale and the PHQ‐9 to screen for depression and anxiety, clinicians should consider adding suicide‐specific screening questionnaires such as the Columbia‐Suicide Severity Rating Scale and the National Institute of Mental Health (NIMH) and the Substance Abuse and Mental Health Services Administration (SAMHSA) Ask‐Suicide Screening Questions, particularly for high‐risk individuals. Both questionnaires are freely accessible through the NIMH or SAMHSA websites. Providers should ask direct questions to patients such as “what are you most worried about?,” “who do you have for support?,” “are you having thoughts of harming yourself right now?,” and “what are your hopes for the future?”^10^

The American College of Obstetricians and Gynecologists recommends that postpartum care be an ongoing process with services tailored to individual needs. The comprehensive postnatal visit, recommended by 12 weeks postpartum, should include a complete assessment of physical, psychological, and social well‐being.^14^ Individuals at high risk for suicide should be in contact with providers more often than the standard schedules of prenatal and postnatal care allow. To optimize the health of women and infants, postpartum care should be considered as an active and evolving process rather than a single encounter, with services and support tailored to each woman's individual needs.

## CONCLUSION

The marked risk of suicidal behavior during pregnancy and within one year after birth reinforces the need for strategies that effectively identify early signs and enable providers to act in a timely manner for suicide prevention. These findings also underscore the need for targeted evidence‐based interventions and effective policies targeting mental health, substance use, intimate partner abuse, and other risk factors to prevent maternal suicide and enhance maternal health outcomes.^15^ Treatment, including a safety plan, should not only align with national models and recommendations for suicide prevention, but also must include a comprehensive understanding and plan to address behaviors and risks that lead to this tragic loss.

## CONFLICT OF INTEREST

The author has no conflicts of interest to disclose.
